# The Leading Authors in Three High Impact Dermatology Journals

**DOI:** 10.2196/39948

**Published:** 2022-08-23

**Authors:** Waseem Hassan, Mehreen Zafar, Joao Batista Teixeira da Rocha

**Affiliations:** 1 Institute of Chemical Sciences University of Peshawar Peshawar Pakistan; 2 Programa de Pós-Graduação em Bioquímica Toxicológica Departamento de Bioquímica e Biologia Molecular Universidade Federal de Santa Maria Santa Maria Brazil

**Keywords:** JAAD, Scopus, bibliometry, dermatology, research, publications, articles, bibliometrics

Recently in dermatology, the most influential scientists were reported [1,2]. Similarly, after analyzing the publications over 10 years (2011-2020), the top authors in the *Journal of the American Academy of Dermatology* *(JAAD)* were also described [3]. In this letter, we are reporting for the first time the top three authors in three world-class journals (ie, JAAD, *JAMA Dermatology* [JAMA-D], and *American Journal of Clinical Dermatology* [AJCD]). On July 24, 2022, the data was retrieved from the Scopus database, and the analysis was performed on RStudio (Bibliometrix/Biblioshiny) software (RStudio, PBC). We only analyzed research articles and excluded the year 2022. Scopus has been covering JAAD, JAMA-D and AJCD since 1979, 2013 and 2000, respectively. In total:

- JAAD published 17,065 research articles. A total of 93 authors published at least 30 articles (for a total of 2732 articles). In these publications, the authors were from 2307 universities in 65 countries.
- JAMA-D has published 1200 articles, of which 5975 authors from 1900 universities in 56 countries have contributed.
- AJCD has published 492 articles. In all publications, 1866 authors were from 838 universities in 45 countries.

During the analysis, authors were included from JAMA-D and AJCD if they had published at least 5 research articles.

There are several bibliometric indicators that can be used for analysis. For example, the h-index considers both the number of publications and citations. In other words, a dermatologist with 5 articles with 5 or more citations (each) will have an h-index of 5. The g-index is another interesting indicator. It represents the highest number “g” of articles that together received g^2^ or more citations. A g-index of 10 indicates that the top 10 publications have been cited at least 100 times (10^2^). The m-quotient (or m-index) considers both the h-index and the number of years. The m-index can be obtained by dividing the h-index by the number of years since the first publication of an author. For example, an h-index of 10 for an individual over 5 years means that the m-quotient is 2.

Our results differed from earlier reports [1-3], as they ranked the authors on the basis of the total number of publications and the h-index. We highlighted the most influential authors in all 3 journals on the basis of the total number of publications, total citations, h-index, g-index, and m-index. The data is presented in Table 1. We also provide the list of the top 10 most productive universities and countries (Table 2).

The major limitations of this letter are name changes (variations in initials) and duplication of common names (authors and universities), which are not addressed. This may affect the ranking. We only relied on Scopus; other databases were not included in this study.

**Table 1:** The list of the top 3 authors in JAAD, AJCD, and JAMA-D.^a^

Number of publications		Total citation	H-index	G-index	M-index
**JAAD^b,c^**					
** **	Feldman SR (n=127)	Feldman SR (n=12,778)	Lebwohl M (55)	Feldman SR (112)	Gelfand JM (1.895)
** **	Lebwohl M (n=108)	Lebwohl M (n=10,127)	Feldman SR (48)	Lebwohl M (100)	Patel KR (1.8)
** **	Paller AS (n=77)	Menter A (n=7754)	Gottlieb AB (39)	Paller AS (77)	Silverberg JI (1.769)
**AJCD^d,e^**					
** **	Piérard GE (n=10)	Silverberg JI (n=260)	Silverberg JI (8)	Piérard GE (10)	Simpson EL (1.75)
** **	Silverberg JI (n=9)	Armstrong AW (n=244)	Armstrong AW (8)	Silverberg JI (9)	Chen Z (1.25)
** **	Armstrong AW (n=9)	Feldman SR (n=218)	Feldman SR (7)	Armstrong AW (9)	Paller AS (1.25)
**JAMA-D^f,g^**					
** **	Mostaghimi A (n=30)	Margolis DJ (n=1159)	Armstrong AW (15)	Mostaghimi A (24)	Mostaghimi A (2)
** **	Marghoob AA (n=21)	Schmults CD (n=1088)	Margolis DJ (14)	Marghoob AA (21)	Armstrong AW (1.5)
** **	Margolis DJ (n=20)	Weinstock MA (n=1067)	Marghoob AA (14)	Margolis DJ (20)	Tkachenko E (1.5)

^a^The ranking is based on the number of publications, total citations, h-index, g-index, and m-index. The data was retrieved from Scopus on July 28, 2022, and analyzed on RStudio (Bibliometrix/Biblioshiny).

^b^JAAD: *Journal of the American Academy of Dermatology.*

^c^Total publications: 26,185; total citations: 566,620 (citations of 20,000 documents); total h-index: 242 (of 20,000 documents); Impact Factor (2021): 15.48; CiteScore: 8.1; Scientific Journal Ranking (SJR; 2021): 1.948; Source-normalized Impact per Paper (SNIP; 2021): 2.512.

^d^AJCD: *American Journal of Clinical Dermatology*.

^e^Total publications: 1418; total citations: 46,803; total h-index: 99; Impact Factor (2021): 6.23; CiteScore: 12.1; SJR (2021): 1.956; SNIP (2021): 3.024.

^f^JAMA-D: *JAMA Dermatology*.

^g^Total publications: 3544; total citations: 46,377; total h-index: 82; Impact Factor (2021): 11.8; CiteScore: 12.5; SJR (2021): 2.412; SNIP (2021): 3.658.

**Table 2:** List of the top 10 most productive universities and countries.

Universities		Publications, n	Country	Publications, n
***Journal of the American Academy of Dermatology*** **(top 10 universities and countries)**				
** **	Mayo Clinic	171	United States	2395
** **	Northwestern University Feinberg School of Medicine	162	Italy	155
** **	Harvard Medical School	144	Canada	146
** **	University of Pennsylvania	141	Germany	118
** **	Icahn School of Medicine at Mount Sinai	134	Israel	88
** **	University of Pennsylvania Perelman School of Medicine	131	Austria	65
** **	University of California, San Francisco	126	United Kingdom	64
** **	Wake Forest School of Medicine	113	Japan	58
** **	Massachusetts General Hospital	110	France	55
** **	Memorial Sloan-Kettering Cancer Center	87	Spain	45
***American Journal of Clinical Dermatology*** **(top 10 universities and countries)**				
** **	Harvard Medical School	94	United States	778
** **	University of Pennsylvania	74	France	96
** **	Brigham and Women's Hospital	73	United Kingdom	78
** **	University of California, San Francisco	64	Germany	71
** **	University of Pennsylvania Perelman School of Medicine	57	Spain	65
** **	Northwestern University	56	Australia	62
** **	Northwestern University Feinberg School of Medicine	54	Canada	57
** **	Memorial Sloan-Kettering Cancer Center	48	Italy	50
** **	Massachusetts General Hospital	40	Netherlands	46
** **	Inserm	37	Denmark	43
***JAMA Dermatology*** **(top 10 universities and countries)**				
** **	Northwestern University Feinberg School of Medicine	17	United States	206
** **	Icahn School of Medicine at Mount Sinai	15	Germany	46
** **	Centre Hospitalier Universitaire de Liege	13	Italy	37
** **	University of Texas MD Anderson Cancer Center	11	Turkey	35
** **	University of Toronto	11	Canada	33
** **	Friedrich-Schiller-Universität Jena	11	United Kingdom	29
** **	Springer Nature	11	France	24
** **	Ankara Numune Education and Research Hospital	10	New Zealand	24
** **	Harvard Medical School	10	Belgium	19
** **	University of Southern California	10	Spain	18

## Abbreviations

AJCD: American Journal of Clinical Dermatology
JAAD: Journal of the American Academy of Dermatology
JAMA-D: JAMA Dermatology
